# Sigma-2 Receptor/TMEM97 and PGRMC-1 Increase the Rate of Internalization of LDL by LDL Receptor through the Formation of a Ternary Complex

**DOI:** 10.1038/s41598-018-35430-3

**Published:** 2018-11-15

**Authors:** Aladdin Riad, Chenbo Zeng, Chi-Chang Weng, Harrison Winters, Kuiying Xu, Mehran Makvandi, Tyler Metz, Sean Carlin, Robert H. Mach

**Affiliations:** Department of Radiology, University of Pennsylvania, Philadelphia, PA USA

## Abstract

CRISPR/Cas gene studies were conducted in HeLa cells where either PGRMC1, TMEM97 or both proteins were removed via gene editing. A series of radioligand binding studies, confocal microscopy studies, and internalization of radiolabeled or fluorescently tagged LDL particles were then conducted in these cells. The results indicate that PGRMC1 knockout (KO) did not reduce the density of binding sites for the sigma-2 receptor (σ2R) radioligands, [^125^I]**RHM-4** or [^3^H]DTG, but a reduction in the receptor affinity of both radioligands was observed. TMEM97 KO resulted in a complete loss of binding of [^125^I]**RHM-4** and a significant reduction in binding of [^3^H]DTG. TMEM97 KO and PGRMC1 KO resulted in an equal reduction in the rate of uptake of fluorescently-tagged or ^3^H-labeled LDL, and knocking out both proteins did not result in a further rate of reduction of LDL uptake. Confocal microscopy and Proximity Ligation Assay studies indicated a clear co-localization of LDLR, PGRMC1 and TMEM97. These data indicate that the formation of a ternary complex of LDLR-PGRMC1-TMEM97 is necessary for the rapid internalization of LDL by LDLR.

## Introduction

The sigma receptors represent a family of proteins whose function in cell biology is poorly understood. The current classification lists two types of sigma receptors, sigma-1 (σ1) and sigma-2 (σ2) receptors (R)^1–3^. The sigma receptors were identified in 1976 and were initially classified as a member of the opiate receptors^4^. Subsequent studies revealed that they represented a discrete family of receptors. For many years, the sigma receptors were described pharmacologically through the binding of the radioligands [^3^H](+)−pentazocine and [^3^H]DTG. [^3^H](+)−Pentazocine has a high affinity for σ1R whereas [^3^H]DTG bind with equal affinity to both σ1 and σ2 receptors. The σ1R was purified, sequenced and cloned from guinea pig brain in 1996, and bears little sequence homology to any known mammalian receptor^5^. The crystal structure of the σ1R was reported in 2016, and the reported structure shows it as a trimeric species with a single transmembrane spanning region^6^. The σ1R is primarily localized in the endoplasmic reticulum (ER), specifically at the mitochondria-associated ER membrane (MAM)^7^. At the MAM, the σ1R acts as a molecular chaperone for the translocation of inositol-requiring enzyme-1 (IRE-1) in transmitting ER stress signals to the nucleus. The σ1R can also undergo translocation from the ER to the plasma membrane, where it has been found to be associated with a variety of receptors, channels and kinases. This association with a variety of plasma membrane-bound receptor is responsible for the diverse number of biological and pharmacological properties associated with the σ1R.

Although much has been learned about the biology of the σ1R in the two decades since its sequencing and cloning, the same cannot be said of the σ2R. In 2011, Xu *et al*. reported that the σ2R represented a binding site in a protein complex containing Progesterone Membrane Binding Component-1 (PGRMC1)^8^. The association of the σ2R with PGRMC1 was initially made via the photoaffinity tagging of this protein with an azido compound having a high affinity for the σ2R. This study did not conclude the σ2R to be PGRMC1, but reported that altering expression levels of PGRMC1 changed the pharmacological properties of small molecules interacting with the σ2R. Evidence supporting this conclusion was reported in two subsequent papers: Izzo *et al*. reported that knocking down PGRMC1 reduced Aβ oligomer synaptic dysfunction in cultured neurons, and this effect could also be achieved pharmacologically with an antagonist of the σ2R^9^. Secondly, Yi *et al*. reported that genetic knockout or knockdown of *vem-1*, the nematode ortholog of PGRMC1, diminished the neurotoxicity of amyloid precursor protein in a nematode model of AD, and this effect could be demonstrated pharmacologically by using σ2 R antagonists^10^. Collectively, these data suggested a strong association between the σ2R and PGRMC1. PGRMC1 has been proposed to be a membrane-associated progesterone receptor, but its role in cell biology is also poorly understood^11^. PGRMC1 is similar to the σ1R in that it too is thought to be a molecular chaperone, in this case one that is involved in the translocation of heme and lipophilic molecules such as cholesterol and other steroids from the plasma membrane and the ER, mitochondria and other organelles^12,13^. The high association of PGRMC1 and σ2R in breast cancer, and the suspected role of both proteins in cell proliferation, also support an association between these two proteins^14–16^.

Evidence disputing the link between the σ2R and PGRMC1 was first reported by Abate *et al*., who demonstrated that stable knockdown of PGRMC1 or the overexpression of this protein in MCF7 cells did not alter the binding of [^3^H]DTG^17^. A subsequent paper by Chu *et al*. provided similar results in NSC34 cells where PGRMC1 had been genetically knocked out using CRISPR gene editing techniques^18^. In each case, the authors concluded that the σ2R was not PGRMC1 and suggested that the data reported by Xu *et al*. was incorrect^17,18^. However, the original study did not claim that the σ2R was PGRMC1, only that the σ2R was a binding site located in the complex of proteins that contained PGRMC1^8^. Subsequently, a recent report by Alon *et al*. concluded that the protein TMEM97 was not only a [^3^H]DTG binding site, but represented the gene for the σ2R^19^, raising further doubt about the association between the σ2R and PGRMC1^20,21^.

TMEM97 is a protein that has generated interest lately because of its association with lysosomal cholesterol transporter NPC1, a protein whose loss results in Niemann–Pick disease type C1, a fatal lysosomal storage disorder^19^. TMEM97 has also been associated with the LDL receptor, and a previous study has shown that siRNA studies knocking down TMEM97 reduced the rate of internalization of LDL by the LDL receptor^22^. Prior to its identification as TMEM97, the σ2R had also been suggested to be involved in cholesterol biosynthesis^13^. Consequently, the association of TMEM97/σ2R, the new nomenclature for this protein, with cholesterol pathways was consistent with prior literature reports on the potential role of the σ2R in cell biology. Since PGRMC1 has also been implicated with cholesterol trafficking (reviewed in^13^), there is a distinct possibility that both TMEM97/σ2R and PGRMC1 may be involved in the same biochemical pathways within the cell. Therefore, as part of our continued interest in the role of the σ2R in cell proliferation, our group conducted a series of CRISPR studies aimed at exploring the functional relationship between TMEM97/σ2R and PGRMC1 on the mechanisms of cholesterol uptake and translocation in the cell. In this report, we present evidence demonstrating that deletion of either TMEM97/σ2R or PGRMC1 reduces the level of internalization and trafficking of LDL by the LDL receptor. Knocking out both proteins in HeLa cells did not have an additive effect on LDL uptake. This effect could also be produced through the pharmacological blocking of TMEM97/σ2R with a σ2R antagonist. These data indicate that *both TMEM97/*σ*2R and PGRMC1* are involved in the internalization of LDL by LDL receptor. Confocal microscopy studies also show that LDLR, PGRMC1 and TMEM97 have an asymmetric distribution in the plasma membrane, suggesting that they are involved in a protein complex. We also report that CRISPR gene editing of TMEM97/σ2R results in a complete reduction of the binding of the σ2R ligand [^125^I]*N*-(4-(6,7-dimethoxy-3,4-dihydroisoquinolin-2(1 H)-yl)butyl)-2,3-dimethoxy-5-iodo-benzamide ([^125^I]**RHM-4**)^23^, but only a partial reduction in the specific binding of [^3^H]DTG to HeLa cell membranes. The residual binding of [^3^H]DTG was not attributed to binding to the σ1R, and may represent a third, currently uncharacterized sigma receptor.

## Results

### CRISPR Gene Editing of PGRMC1 and TMEM97

**Figure 1:**
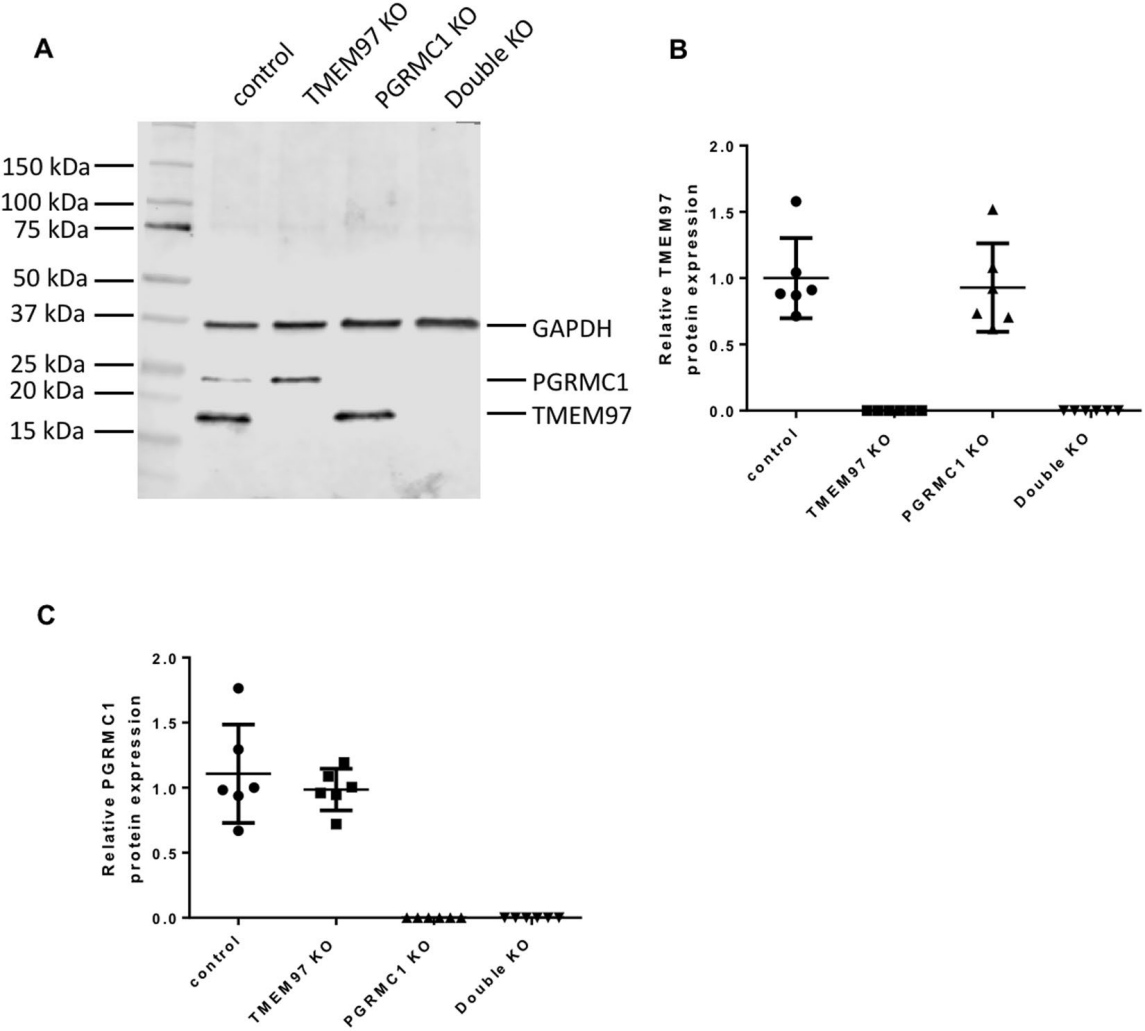
Western blot analysis for control, TMEM97 KO, PGRMC1 KO, and double KO cell lines. All the four cell lines were generated by sequential plasmid transfection and lentivirus-infection procedures. Single clones were isolated for all cell lines. (**A**) A representative western blot showing that TMEM97 or/and PGRMC1 were completely deleted in the knockout cell lines. (**B**) TMEM97 and (**C**) PGRMC1 protein levels measured as the expression ratio of TMEM97 or PGRMC1 to GAPDH, respectively, were quantified in control and knockout cell lines. Six clones from each cell line were analyzed. The data showed that TMEM97 KO did not affect the PRGMC1 protein level and vice versa.

In order to study whether TMEM97 or/and PGRMC1 is responsible for σ2R binding and what their biological functions are, CRISPR technology was used to knockout TMEM97, PGRMC1 and both TMEM97 and PGRMC1 in HeLa cells to generate four knockout (KO) cell lines, i.e. control, TMEM97 KO, PGRMC1 KO, and TMEM97/PGRMC1 double KO. All the four cell lines were generated using the same plasmid transfection and lentivirus-infection procedures. Single clones were isolated for all knockout cell lines and used throughout the current study. Western blot showed that TMEM97 or/and PGRMC1 were completely removed in the knockout cell lines (Fig. 1A). TMEM97 or PGRMC1 protein levels were measured as the expression ratio of TMEM97 or PGRMC1 to GAPDH, respectively, were quantified in control and knockout cell lines. Six clones from each cell line were analyzed. The data showed that TMEM97 KO did not affect PRGMC1 protein level and vice versa (Fig. 1B,C). TMEM97 was knocked out by using three different guide RNA sequences, i.e. sgRNA-1, 2 or 3, each directing cas9 nuclease to cleave distinct, specific site of exon 1 of TMEM97. Western blot analysis showed that TMEM97 proteins were successfully deleted by using each of three sgRNAs (Supplementary Fig. 1). Only sgRNA-1 was used to generate TMEM97 knockout cells for the majority of the studies described below.

### *In vitro* Binding Studies

**Figure 2:**
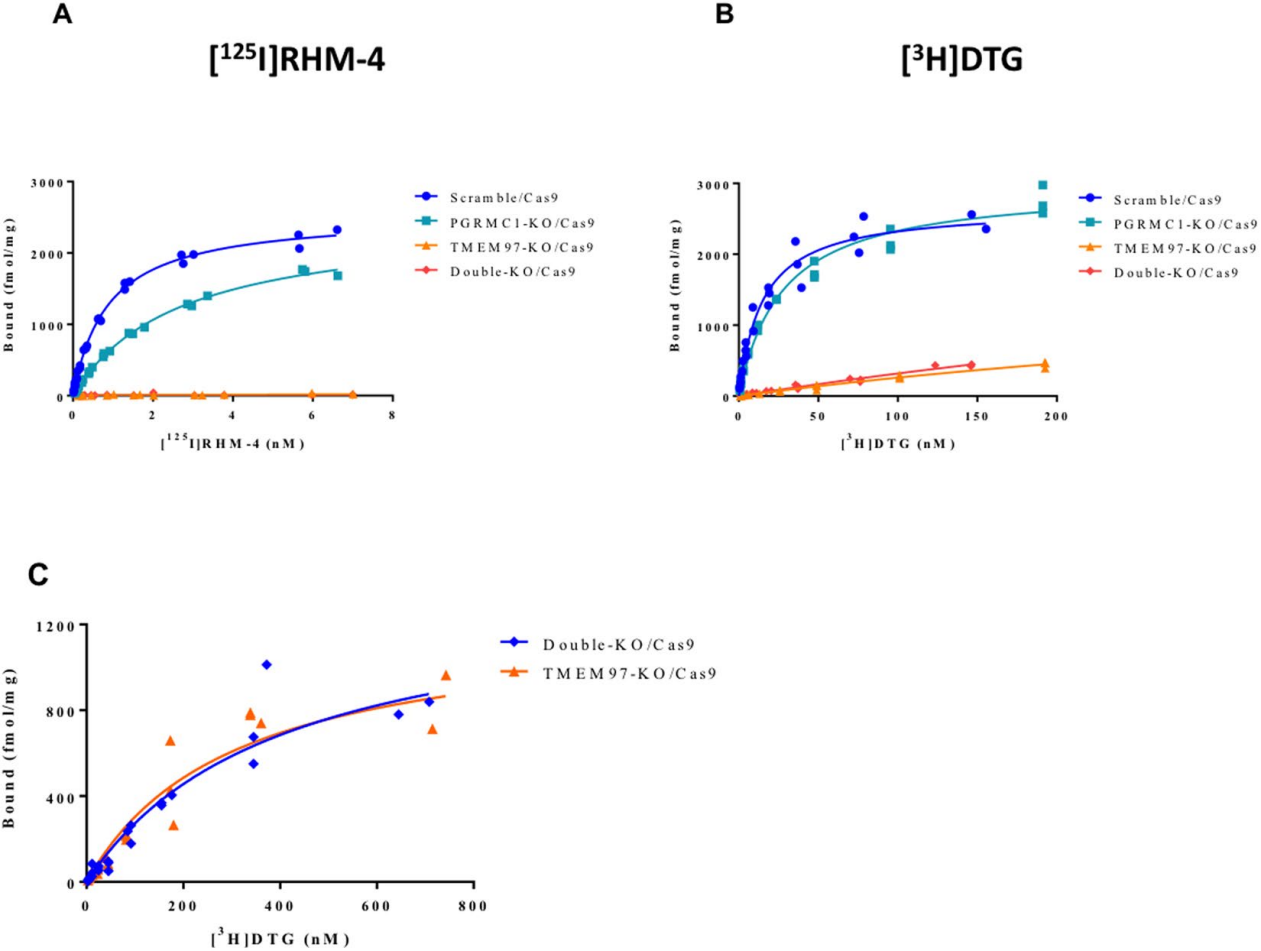
*In vitro* binding studies in control, TMEM97 KO, PGRMC1 KO, and double KO cell lines. (**A**) [^125^I]**RHM-4** binding was completely eliminated in the TMEM97 KO and double KO cells. (**B**) [^3^H]DTG binding was significantly reduced in the TMEM97 KO and double KO cells, but some residual specific binding was observed. (**C)** Saturation binding curves were obtained using a ligand concentration of [^3^H]DTG (1 nM - 750 nM) to obtain Kd and Bmax values for the residual binding site in the TMEM97 KO and double KO cells.

*In vitro* binding studies were conducted using two different radioligands for the σ2R, [^125^I]**RHM-4**, which has a high affinity and selectivity for σ2R versus σ1R, and [^3^H]DTG, which binds equally to σ2R and σ1R. The *in vitro* binding studies were done in the presence of 1 μM (+)−pentazocine to mask σ1R. The results of the binding studies in the PGRMC1 KO cells indicated that there was no change in the Bmax values for either [^125^I]**RHM-4** and [^3^H]DTG. However, knocking out PGRMC1 resulted in a small increase in Kd value of both [^125^I]**RHM-4** and [^3^H]DTG (Fig. 2; Table 1). Knocking out TMEM97 resulted in a complete reduction in binding of [^125^I]**RHM-4** to the HeLa cells (Fig. 2B). This effect was observed in the TMEM97 KO cells using all three guide RNA sequences (Supplementary Fig. 2). We also observed a large, but incomplete, reduction of [^3^H]DTG binding to TMEM97 KO cells (Fig. 2B). The reduction in specific binding of [^3^H]DTG was observed in TMEM97 KO cells using all three guide RNA sequences, and all cell lines studied retained a residual [^3^H]DTG binding capacity. To better characterize the residual DTG binding site, saturation ligand binding studies were performed on TMEM97 KO cells using [^3^H]DTG (Fig. 2C). We determined a Kd value for this residual DTG binding site to be 302 ± 80 nM with a Bmax value of 1218 ± 148 fmol/mg protein.

**Table 1:** *In vitro* binding data from the radioligand binding studies.

	Kd (nM)		B_max_ (fmol/mg)	
	[^3^H]DTG	[^125^I]RHM-4	[^3^H]DTG	[^125^I]RHM-4
Scramble/Cas9	14.8 ± 1.8	0.88 ± 0.04	2664 ± 105	2545 ± 37
PGRMC1 KO/Cas9	27.5 ± 2.5	2.66 ± 0.12	2971 ± 91	2475 ± 51
TMEM97 KO/Cas9	302.0 ± 80.2	N.B.	1218 ± 148	N.B.
Double KO/Cas9	402.6 ± 109.8	N.B.	1374 ± 192	N.B.

N.B. = no binding.

Previous studies have reported that [^3^H]DTG binds with low affinity to the PGRMC1 homodimer in cells overexpressing this protein^24^. In order to confirm that this residual binding site did not reflect binding to the PGRMC1 homodimer, *in vitro* ligand binding studies were conducted in the double KO cells (Fig. 2C). We observed no significant difference in the [^3^H]DTG Bmax and Kd between the TMEM97 KO and the TMEM97/PGRMC1 double KO cells (Table 1), indicating that the residual binding of [^3^H]DTG is not related to PGRMC1.

Cancer cells have also been reported to possess splice variants of the σ1R that have low affinity for [^3^H](+)-pentazocine and [^3^H]DTG^25^. In order to confirm that the residual [^3^H]DTG binding site did not reflect binding to splice variants of the σ1R, Western blots were obtained for the HeLa cells used in this study using an antibody that recognizes all forms of the σ1R. The results of this study revealed that the HeLa cells used in this study contained only the full length σ1R (Data not shown). Therefore, this residual [^3^H]DTG binding site does not represent binding to a σ1R splice variant.

### Interactions between TMEM97, PGRMC1, and the LDL Receptor

Cholesterol, due to its hydrophobic nature, is transported in the bloodstream bound with LDL and is taken up into cells primarily via the binding of LDL to the LDLR followed by receptor-mediated endocytosis^26^. Since both TMEM97 and PGRMC1 are membrane-bound proteins that have been implicated in cholesterol metabolism, a series of studies were conducted to determine if there is a possible interaction between these proteins and the internalization of LDL by the LDLR. There was no change in protein expression levels of LDLR between the control (Scramble/Cas9), PGRMC1 KO, TMEM97 KO, and double KO cells as assessed by western blot. TMEM97 was not detected in the TMEM97 KO and double KO cell lines, and PGRMC1 was not detected in the PGRMC1 KO cells and double KO cell lines, verifying the knockout of the representative proteins (Fig. 1; Supplementary Fig. 3). Total intracellular cholesterol levels were not affected in the knockout cell lines as assessed by filipin staining (Supplementary Fig. 4).

**Figure 3:**
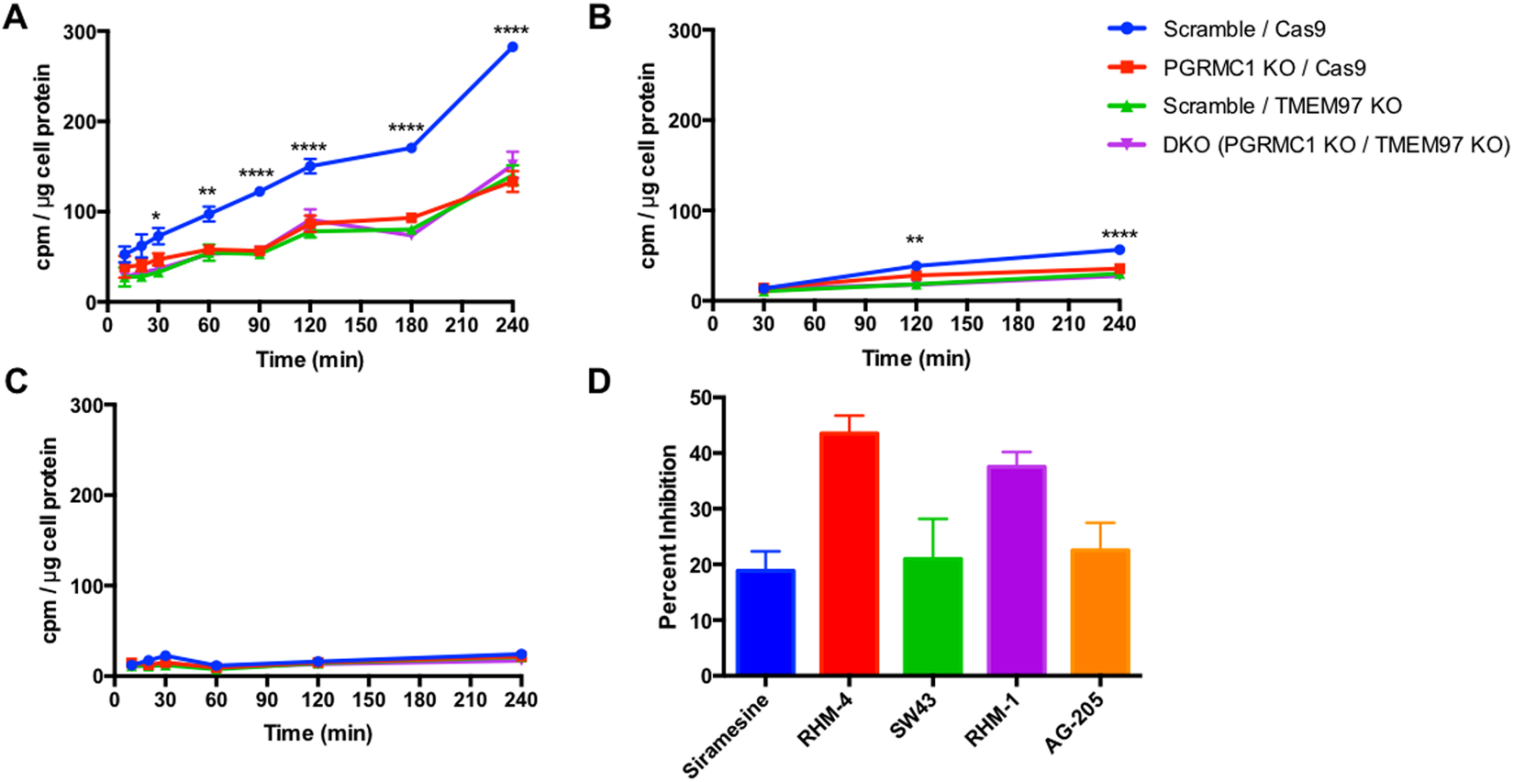
Uptake of [^3^H]cholesterol labeled LDL (50 μg/mL LDL labeled with 3 μCi/mL [^3^H]cholesterol) expressed as cpm /μg cell protein. (**A**) Uptake over time. (**B**) Uptake over time with the addition of excess unlabeled LDL (250 μg/mL). (**C**) Incubation at 4 °C to indicate similar capacity of LDL binding to LDL receptor. (**D**) Percent Inhibition of radiolabeled LDL uptake by incubation with 500 nM Siramesine, RHM-4, SW43, RHM-1, and AG-205. Significance indicated by a 2-way Anova for comparison between cell lines. *Indicates p < 0.05, **p < 0.01, ***p < 0.001, ****p < 0.0001, n = 4.

The next series of experiments studied the effect of knocking out TMEM97 and PGRMC1 on the internalization of LDL by LDLR. Since LDL is internalized via clathrin-mediated endocytosis, we measured the kinetics of LDL internalization using LDL labeled with [^3^H]cholesterol. Cells were incubated in the presence of lipoprotein-depleted serum (LPDS) for 24 hours in order to induce expression of LDLR. As noted above, there was no difference in LDLR expression between the control cells and any of the knockout cell lines (Supplementary Fig. 3). Uptake of radiolabeled LDL was significantly decreased in PGRMC1 KO, TMEM97 KO, and double KO cell lines. Moreover, this decrease in LDL internalization was not a stepwise reduction with the loss of each protein but rather a complete diminished capability, as PGRMC1 KO and TMEM97 KO cells had the same decreased internalization of radiolabeled LDL as the double KO cells (Fig. 3A). This decreased LDL uptake was also observed in experiments using fluorescently tagged DiI-LDL (Supplementary Fig. 5). To ensure the radiolabeled LDL was taken up via the LDLR pathway, excess unlabeled LDL (250 μg/mL) was incubated with the radiolabeled LDL (50 μg/mL). Under these conditions the excess LDL competes with radiolabeled LDL for binding with LDLR, resulting in significantly decreased internalization (Fig. 3B). These data confirm that the LDL-LDLR pathway is responsible for internalization in these cells.

The next set of experiments was conducted to identify if PGRMC1 and TMEM97 are responsible for the initial binding of LDL to the LDLR, or if they are important for the internalization of the bound LDL-LDLR complex. Incubation of HeLa cells in the presence of LDL at 4° allows LDL to bind with LDLR but inhibits receptor-mediated endocytosis of the bound complex^27^. We observed no significant difference between knockout and control cells in their capacity to bind LDL (Fig. 3C). Their similar capacity to bind LDL further suggests that both PGRMC1 and TMEM97 are involved in the internalization of the LDL-LDLR complex, and they are not involved in the initial binding of LDL to LDLR.

The next logical step was to evaluate the effect of pharmacological inhibition of TMEM97 on the uptake of LDL and compare it to ablation of the protein via knockout. Since TMEM97 is the protein which contains the σ2R, we used two ligands previously described selective σ2R antagonists **RHM-4** (Kd ~ 0.2 nM) and RHM-1 (Kd ~ 0.66 nM); two ligands previously described as being σ2R agonists **Siramesine** (Kd ~ 1.6 nM) and **SW43** (Kd ~ 12 nM). We also used AG-205 a PGRMC1 inhibitor (Kd ~ 1 μM against σ2R and Ki~1263 ± 325 to the σ2R for displacing DTG). All compounds resulted in a reduction of LDL uptake, with the classical σ2R antagonists having a greater effect than both the σ2R agonists and the PGRMC1 inhibitor. There was a dose-dependent reduction in uptake of LDL labeled with [^3^H]cholesterol when HeLa cells were treated with these inhibitors (Supplementary Fig. 6). This pharmacologic inhibition of LDL uptake was observed in control cells only, but did not further decrease uptake in the knockout cells lines, further suggesting that PGRMC1 and TMEM97 must both be present and functional for effective LDL-LDLR complex internalization (Supplementary Fig. 7).

### Somatostatin and Insulin Uptake Rates are unaffected in knockout cell lines

To determine whether the presence of TMEM97 and/or PGRMC1 affects all clathrin-mediated endocytic pathways, we examined at the uptake of radiolabeled [^125^I]TYR^11^-Somatostatin and [^125^I] Insulin. Results indicated that there was no difference between the rate of uptake of somatostatin or insulin between the cell lines (Supplementary Fig. 8). This indicates that TMEM97 and PGRMC1 play a unique role in uptake of LDL via their interaction with LDLR.

### Confocal Microscopy Studies

**Figure 4:**
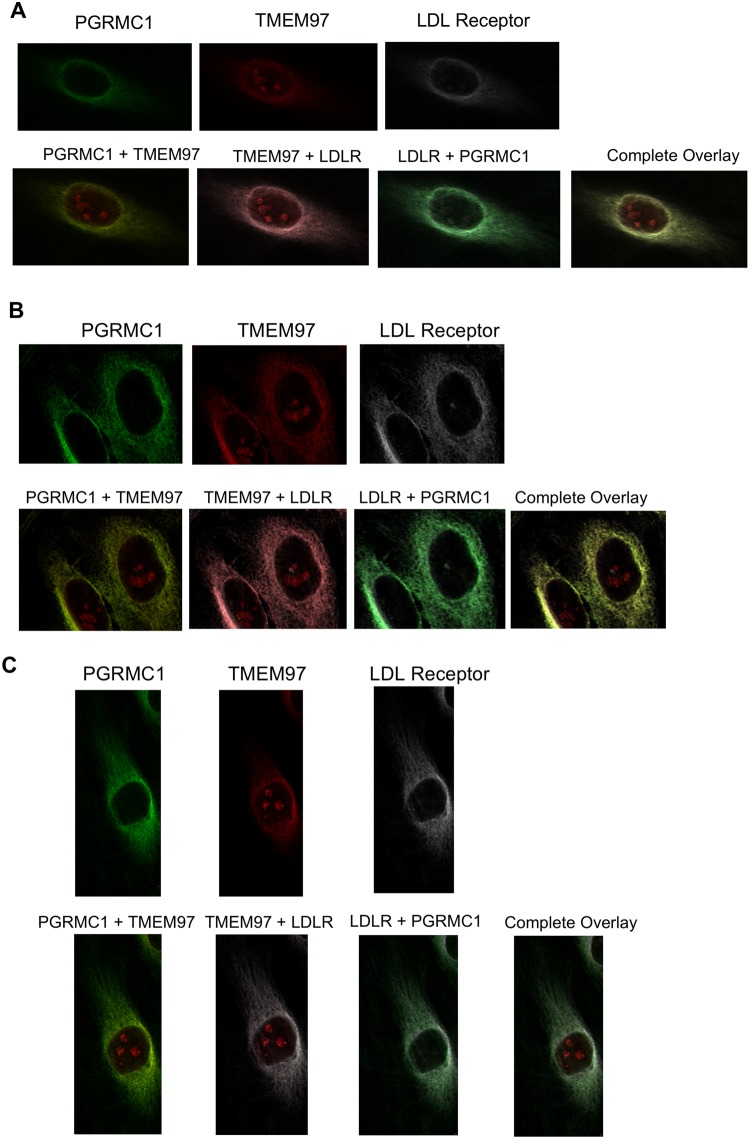
Confocal microscopy on HeLa cell lines labeled with antibodies against LDLR, PGRMC1, and TMEM97. (**A**) Cells were plated and imaged under standard, non-lipoprotein depleted conditions. (**B**) Cells were plated and sterol starved by incubation with lipoprotein-depleted serum for 24 hours. (**C**) Cells were sterol starved for 24 hours, followed by treatment with 50 μg/mL LDL for 3 hours.

A series of confocal microscopy studies was conducted in order to determine the cellular localization of TMEM97, PGRMC1 and LDLR in HeLa cells. Under serum-starved conditions, there was an asymmetrical distribution of LDLR in the plasma membrane of HeLa cells. This asymmetry of LDLR has been reported previously and is thought to represent localization of LDLR in the region of the plasma membrane rich in lipid rafts. There was a similar asymmetric localization of both TMEM97 and PGRMC1 in the plasma membrane. Co-localization studies indicate that there is a high degree of co-localization of LDLR, TMEM97 and PGRMC1 under these conditions. However, there was a population of PGRMC1 sites that were not co-localized with LDLR and TMEM97 (Fig. 4, Supplementary Fig. 9).

The next set of experiments repeated the confocal imaging studies, this time following treatment of previously serum-starved HeLa cells with serum containing LDL. Although there was an asymmetric distribution of LDLR, TMEM97 and PGRMC1 in HeLa cells, and co-localization of the three proteins, there was also a higher percentage of both PGRMC1 and TMEM97 not associated with LDLR. These data are consistent with the dissociation of the LDL-LDLR-TMEM97-PGRMC1 protein complex following internalization into the lysosomal compartments within the cell. The confocal microscopy data, taken collectively with the cell uptake studies of [^3^H]cholesterol-labeled LDL and fluorescently-tagged LDL, suggest that the rapid internalization of LDL by the LDLR requires the formation of a complex with both PGRMC1 and TMEM97.

### Proximity Ligation Assays

**Figure 5:**
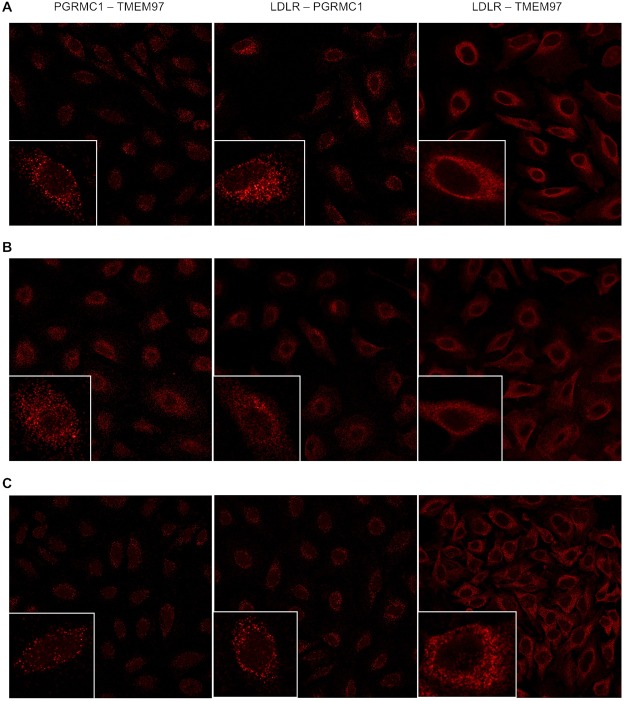
Proximity Ligation Assay on Scramble/Cas9 HeLa cells showing interactions between TMEM97 and PGRMC1, LDLR and PGRMC1, and LDLR and TMEM97. (**A**) standard, non-lipoprotein depleted conditions, (**B**) sterol-starved conditions, and (**C**) sterol-starved conditions followed with treatment with 50 μg/mL LDL for 3 hours. Inserts represent an enlarged region focusing on a single cell.

To confirm that the observed associations between the protein was indeed a complex formed, proximity ligation assays were conducted pairwise between LDLR, PGRMC1, and TMEM97. We observed interactions between LDLR, TMEM97, and PGRMC1 (Fig. 5) in normal, sterol-starved, and sterol-starved followed by LDL treatment for 3 hours. The interactions between LDLR and TMEM97 and between TMEM97 and PGRMC1 were not observed in the TMEM97 KO and double KO cell lines (Supplementary Fig. 10), as expected. Similarly, the interactions between LDLR and PGRMC1 and between TMEM97 and PGRMC1 were not observed in the PGRMC1 KO and double KO cell lines. However LDLR and PGRMC1 were shown to interact in the TMEM97 KO, and between LDLR and TMEM97 in the PGRMC1 KO, indicating that the LDLR interacts with TMEM97 and PGRMC1 despite the absence of the other protein.

Taken together this data indicated that the LDLR, TMEM97, and PGRMC1 proteins are associated within close proximity of each other and form a complex.

## Discussion

The goal of the current study was to further understand the role of the σ2R in cell biology, particularly its role as a molecular marker of cell proliferation. Previous studies from our group have shown that the density of σ2Rs is higher in proliferating breast cancer cells relative to cancer cells driven into quiescence (i.e., G0) by prolonged serum starvation^28,29^. PET imaging studies with a σ2R -selective radiotracer, [^18^F]ISO-1, have shown a strong correlation between radiotracer uptake and the density of σ2R in a murine model of breast cancer^30^, and an excellent correlation between radiotracer uptake and Ki-67 score in a mixed population of patients diagnosed with lymphoma, breast, and head & neck cancer^31^. Since the molecular identity of the σ2R was not known at the time of these studies, these encouraging results initiated a series of studies in our lab to characterize this protein as a means of understanding the role of the σ2R in cell proliferation. In 2011, we reported a series of experimental results suggesting that the σ2R was a binding site in a protein complex containing PGRMC1^8^. We did not state that the σ2R was PGRMC1 since we did not observe a 1:1 correlation between the binding of a radioiodinated ligand having a high affinity for the σ2R, and levels of the protein in HeLa cells having either a transient knockdown or overexpression of PGRMC1^8^. Subsequent studies using cancer cells having a stable knockdown or overexpression of PGRMC1 challenged these conclusions since neither condition resulted in a change in binding of the σ2R radioligand, [^3^H]DTG^17,18^. The recent identification of the σ2R as being the protein, TMEM97, provided further support to the absence of any correlation between the σ2R, now called the TMEM97/σ2R, and PGRMC1^19,21^.

TMEM97 is thought to be involved in cholesterol trafficking through its association with lysosomal cholesterol transporter NPC1, a protein whose loss results in a fatal lysosomal storage disorder, Niemann–Pick disease type C1^19^. A previous study also demonstrated that siRNA studies knocking down TMEM97 reduced the rate of internalization of LDL by the LDL receptor (LDLR)^22^. Another protein that has been strongly linked to cholesterol trafficking is PGRMC1, and in a recent review by Cahill and Medlock, a theoretical mechanism showing a role of PGRMC1 in the membrane trafficking of LDLR was proposed^32^. Therefore, if the σ2R and PGRMC1 are part of a protein complex as we proposed in 2011, the above evidence suggests that this protein complex may consist of the LDLR, TMEM97 and PGRMC1.

In the current study, we provide strong experimental evidence that this is indeed the case. CRISPR gene editing studies resulting in a complete knockout of either TMEM97 or PGRMC1 resulted in an identical reduction in the rate of internalization of ^3^H-labeled LDL and fluorescently tagged DiI-LDL by LDLR. Knocking out both TMEM97 and PGRMC1 did not lead to a further reduction in ^3^H-LDL uptake, indicating that the effect was not additive, and loss of one protein has the same effect as losing both proteins. The decreased uptake in the absence of TMEM97, PGRMC1, or both was unique to LDL, as uptake of somatostatin and insulin was not affected, thus demonstrating that knocking out these proteins does not disrupt all clathrin-mediated endocytic pathways. Furthermore, knocking out TMEM97 resulted in a complete loss of binding of [^125^I]**RHM-4**, and a significant reduction in the binding of [^3^H]DTG to HeLa cells. These data indicate that [^125^I]**RHM-4** binds selectively to TMEM97, whereas there is some residual binding of [^3^H]DTG to a site not associated with TMEM97. The affinity of [^3^H]DTG for this residual binding site is ~300 nM, and its density is ~40% that of TMEM97. Since this residual binding was also observed in the double KO cells, it does not represent binding to the low affinity DTG binding site identified previously in the PGRMC1 dimer. Whether this site represents a third, previously unidentified sigma receptor or some other off-target binding site remains to be determined. It is also interesting to note that knocking out PGRMC1 resulted in a decrease in affinity of both [^125^I]**RHM-4** and [^3^H]DTG for TMEM97, but no change in Bmax values for the protein. These results are consistent with an interaction between TMEM97 and PGRMC1, with the knocking out of PGRMC1 resulting in a small change in the 3-dimensional structure of TMEM97 leading to a reduction in affinity of σ2R radioligands binding to this site.

The confocal microscopy and proximity ligation assay studies reported here also support the above conclusions. There was an asymmetric distribution of LDLR, TMEM97, and PGRMC1 in the plasma membrane of HeLa cells under serum-starved conditions^33,34^. Co-localization studies indicate that there is a high level of co-localization of the three proteins, which is consistent with the formation of an LDLR-TMEM97-PGRMC1 protein complex. However, there was also a population of PGRMC1 apparently not associated with LDLR, as evident in the proximity ligation assay, where the PGRMC1-TMEM97 and LDLR-PGRMC1 pairs showed less colocalization signal than the LDLR-TMEM97 pair. These data are consistent with previous reports that PGRMC1 may be associated with other membrane-bound receptors such as the epidermal growth factor receptor (EGFR) and insulin receptor (IR)^22,35^. Addition of LDL to HeLa cells after a brief period of serum starvation resulted in the internalization and likely dissociation of the LDL-LDLR-TMEM97-PGRMC1 tetrameric complex in the acidic environment of the lysosomes since this treatment resulted in an increase in the amount of PGRMC1 and TMEM97 not co-localized with LDLR.

The involvement of TMEM97/σ2R in the internalization and trafficking of cholesterol via LDL is consistent with our previous characterization of the σ2R is upregulated in proliferating versus quiescent breast cancer cells. Cholesterol is a key component of the cell membrane and an essential requirement for cell division, and an increase in cholesterol uptake has been demonstrated for proliferating cells^36–38^. Since the demand for cholesterol to support cell proliferation is high, most cancer cells are characterized by an upregulation of the various pathways responsible for its biosynthesis^36,39^. A disruption of cholesterol biosynthesis has also been proposed as a potential mechanism for treating cancer^36,38,39^.

The disruption of lipoprotein trafficking may also be responsible for the mechanism of action of σ2R antagonists in preventing Aβ oligomer synaptic dysfunction and Aβ-induced neurotoxicity. Apolipoprotein-E (Apo-E) is the main source of cholesterol in brain, which is required for axonal growth and synaptogenesis. Apo-E is synthesized in astrocytes and microglia and released into the extracellular space where it is taken up in neurons by LDLR and a second lipoprotein receptor, LDL receptor related protein 1 (LRP1). There is a strong correlation between the prevalence of AD and lipoprotein metabolism, in particular apolipoprotein e4 (APO-E4). For example, patients having the APO-E4 allele have a higher incidence of AD and are positive for Aβ plaques as measured by [^11^C]PiB PET imaging studies at an earlier age. Apolipoproteins also bind Aβ, and it has been proposed that Apo-E-bound Aβ is taken up by neurons via transport by LDLR or LRP1. Therefore, it is possible that σ2R antagonists disrupt the binding of Aβ oligomers or Aβ-bound Apo-E to LDLR or LRP1 in a manner analogous to blocking the uptake of LDL in HeLa cells by the σ2R antagonist, **RHM-4**.

In summary, our data demonstrate that the rate of uptake of LDL by LDLR is increased by the formation of a complex with both PGRMC1 and TMEM97. Internalization of LDL by LDLR can occur in the absence of this protein complex, but at a much slower rate than what is observed with the ternary complex. Confocal microscopy and radioligand binding studies indicate that PGRMC1 and TMEM97 are physically associated in this complex with LDLR since knocking out PGRMC1 results in a reduction in the affinity of σ2R ligands for TMEM97, and there is a high degree of co-localization of these proteins. The formation of a trimeric LDLR-PGRMC1-TMEM97 complex that results in the rapid internalization and trafficking of LDL is consistent with the conclusion we stated in our 2011 paper, that the σ2R represents a binding site in the protein complex containing PGRMC1.

## Materials and Methods

*N*-(4-(6,7-dimethoxy-3,4-dihydroisoquinolin-2(1H)-yl)butyl)-2,3-dimethoxy-5-iodo-benzamide and [^125^I]*N*-(4-(6,7-dimethoxy-3,4-dihydroisoquinolin-2(1H)-yl)butyl)-2,3-dimethoxy-5-iodo-benzamide were synthesized as previously described^23,40^. [^3^H]DTG, [1,2-^3^H(N)]cholesterol (49.8 Ci/mmol), [^125^I]TYR^11^-Somatostatin 14 (2200 Ci/mmol), and [^125^I] Insulin (2200 Ci/mmol) were purchased from Perkin Elmer (NET986001MC, NET139001MC, NEX389010UC, and NEX420010UC, respectively).

### PGRMC1 knockout in HeLa cells

PGRMC1 was knocked out in HeLa cells using CRISPR/Cas9 technology. PGRMC1 CRISPR/Cas9 KO Plasmid and control CRISPR/Cas9 Plasmid was purchased from Santa Cruz biotechnology (sc-401945 and sc-418922, respectively). Transfection of the plasmid was conducted according to manufacturer’s instructions. Briefly, HeLa cells were plated in 6-well plates at a cell density of 5 × 10^5^/well. 1 μg of plasmid DNA in 150 μl Plasmid transfection medium was added dropwise to 150 μl of diluted UltraCruz Transfection Reagent (10 μl UltraCruz Transfection Reagent plus 140 μl Plasmid transfection medium). The resulting solution was mixed by vortexing immediately and then incubated for 20 min at room temperature. Prior to transfection, cell culture media was replaced with 2 ml of fresh, antibiotic-free media. Then the 300 μl Plasmid DNA/UltraCruz® Transfection Reagent Complex was added dropwise to the well. 24–48 hr after transfection, successful transfection of CRISPR/Cas9 Knockout Plasmid was visually confirmed by cellular expression of green fluorescent protein (GFP) via fluorescent microscopy. The transfected cells were sorted into a 96-well plate at a density of one cell per well using the BD FACSJazz cell sorter (BD Biosciences). Cells were maintained in HeLa media for 2–4 weeks until a colony was formed from a single cell. Cells from multiple single colonies were then trypsinized and expanded. PGRMC1 knockout for each clone was examined by western blot analysis.

### TMEM97 knockout in HeLa cells

Generating cell lines with stable knockout of TMEM97 was conducted by using a two-vector lentiviral method^1^. Lentivirus was produced by using lentiCas9-Blast (Addgene plasmid # 52962, gift from Feng Zhang)^41^ and three packaging plasmids (pMD2.g/VSVG (plasmid # 12259), pRSV-REV (plasmid # 12253), pMDLg/pRRE (plasmid # 12251), (Addgene, Cambridge, MA) in HEK-293T cells with LipoD293 transfection reagent under standard conditions. Media was changed 6 hr after transfection, and was subsequently collected and filtered 48 hr after transfection using a 0.2 Micron filter syringe unit. Virus was immediately added to cells of interest with 8 μg/mL polybrene for 24 hr. 72 hr after infection, cells were then selected for stable expression of cas9 using 8 μg/mL of blasticidin for one week. Next, knockout populations were created by cloning a sgRNA sequence targeting Exon 1 of TMEM97 into lentiGuide-Puro plasmid (Addgene plasmid #52963, gift from Feng Zhang)^1^. The guide RNA sequence is 5′ CTATGGGGGCTCCGGCAACC 3′.

Lentivirus for the sgRNA plasmid was generated as described previously and used to infect cell lines stably expressing cas9. Cells were then selected for stable expression of sgRNA using 2 μg/mL puromycin for one week. TMEM97 knockout single cell clones were generated using the same method described above for isolating PGRMC1 knockout clones. TMEM97 deletion was confirmed by western blot analysis.

### PGRMC1 and TMEM97 double knockout in HeLa cells

PGRMC1 and TMEM97 double knockout cell line was generated by first knocking out PGRMC1, then knocking out TMEM97 in HeLa cells using the methods described above. The control cell line was generated by two sequential procedures: (1) transfecting HeLa cells with the non-specific 20-nucleotide RNA CRISPR/Cas9 Plasmid (Santa Cruz biotechnology), and (2) transducing the resulting cells with lentivirus containing lentiCas9-Blast plasmid and then selecting cells for stable expression of Cas9. Single cell clones were isolated for all cell lines.

### Western blot analysis

Cells were lysed in radioimmunoprecipitation assay (RIPA) buffer (sc-24948 Sigma-Aldrich, St. Louis, MO, USA) containing 150 mM NaCl, 1.0% IGEPAL^®^ CA-630, 0.5% sodium deoxycholate, 0.1% SDS, 50 mM Tris, pH 8.0 supplemented with protease inhibitor cocktail, and phosphatase inhibitor cocktail 2 and 3 (sc-24948 Sigma-Aldrich, St. Louis, MO, USA). The cells were sonicated briefly, centrifuged at 13,000 × g for 20 min at 4 °C, and the supernatant collected. The protein concentration was determined using a Bio-Rad DC protein assay kit (5000112 Bio-Rad Laboratories, Hercules, CA, USA). Lysates containing 20 µg of protein were run on a 4–20% acrylamide gel and transferred to a PVDF membrane using the Trans-Blot Turbo Transfer System (Bio-Rad Laboratories, Hercules, CA, USA). The PVDF membrane was incubated with Odyssey blocking buffer (927–40100 Licor Biotechnology, Lincoln, NE) for 1 hr at room temperature, then overnight with a rabbit anti-TMEM97 antibody (OAAB22200 Aviva Systems Biology, San Diego, CA) at a 1:8000 dilution, or a rabbit anti-PGRMC1 antibody (HPA002877 Sigma Aldrich, St. Louis, MO) at a 1:1000 dilution at 4 °C, and finally with the secondary antibody, IRDye 800CW anti-rabbit IgG (925–32211 Licor Biotechnology, Lincoln, NE) at a 1:15,000 dilution. The signals were detected and quantified using the Odyssey® CLx Infrared Imaging System (Licor Biotechnology, Lincoln, NE).

### Saturation binding assays

For the cell membrane preparation for receptor binding, the cell pellets were taken out from the freezer, rinsed with 10 mL ice-cold PBS solution, and homogenized by using Wheaton overhead stirrer (120 Vac Overhead Stirrer, Millville, NJ, USA) at the speed of 2 for 30 sec. Then the cell solution was transferred to a 50 mL conical tube and centrifuged for 20 min at 31,000 × g, 4 °C. After centrifugation, the supernatant was discarded and the pellets were resuspended in 1 mL ice-cold PBS solution and stored at −80 °C freezer.

The saturation binding protocols of [^3^H]DTG and [^125^I]RHM-4 were conducted as described below. Briefly, cell membranes were incubated with [^3^H]DTG (0.5–200 nM in 50 mM Tris, pH 8.0) or [^125^I]RHM4 (0.02–9 nM in 50 mM Tris, pH 7.4) for 120 min or 90 min at room temperature, respectively. The nonspecific binding signal for both groups was defined with 10 μM DTG, and 1 μM of (+)−pentazocine was added for [^3^H]DTG binding to mask the σ1R binding site. After incubation, the bound ligands were collected with a M-24 Brandel filtration system (Brandel, Gaithersburg, MD, USA) on the glass fiber papers (Whatman grade 934-AH, GE Healthcare Bio-Sciences, Pittsburgh, PA), and counted with a MicroBeta^2^ Microplate counter 2450 or Wizard2 Automatic Gamma Counter 2470. All the K_d_ and B_max_ values were calculated from a nonlinear regression method with Prism, and the protein concentrations were determined by the method of Lowry *et al*.^4^ with BSA as a standard.

To further evaluate the residual binding of [^3^H]DTG on TMEM97 KO and double KO cell membranes, a concentration of [^3^H]DTG (2–700 nM) was applied for the incubation. The nonspecific binding for these assays was defined with 60 μM DTG, and the σ1R binding of [^3^H]DTG was masked with 5 μM PRE-084 (P2607 Sigma Aldrich, St. Louis, MO) due to its high selectivity for σ1R^42^.

### Preparation of Radiolabelled LDL

Radiolabeled LDL was prepared by incubating human LDL (50 μg/mL) (LP2, Millipore) with [^3^H]cholesterol (final concentration 3 μCi/mL) in serum-free MEM for 24 hours at 37 °C, filtered using a 0.2 μm filter, and used immediately. Care was taken not to vortex LDL to avoid aggregation of particles.

### Radiolabeled LDL Uptake Assay

Cells were plated on 6-well plates (1 × 10^5^ cells per well) or for 96-well plates (1 × 10^4^ cells per well) in MEM with 10% FBS, 100 I.U./ml penicillin, 100 µg/ml streptomycin, 2mM L-glutamine, and 1X MEM non-essential amino acids. After 24 hours, media was removed and cells were incubated with MEM containing 10% lipoprotein-depleted serum (LPDS) (AAJ65182AMG, Fisher Scientific) for an additional 24 hours. Cells were incubated with radiolabeled LDL for 10–240 minutes at 37° for uptake studies (Fig. 3A,C,D) or 4 °C for the LDL surface binding study (Fig. 3B) with or without the presence of excess non-radiolabeled LDL (250 μg/mL), or with RHM-4, Siramesine, SW43, RHM-1, or AG-205 (1 nM - 500 nM). Cells were washed 3 times in PBS, incubated with scintillation fluid overnight, and counts per minute (cpm) were determined using a scintillation counter MicroBeta^2^ Microplate counter 2450.

### Radiolabeled Somatostatin Uptake Assay

Cells were plated in 96-well stripwell plates (1 × 10^4^ cells per well) in MEM with 10% FBS, 100 I.U./ml penicillin, 100 µg/ml streptomycin, 2mM L-glutamine, and 1X MEM non-essential amino acids. After 24 hours, media was removed and cells were incubated with MEM containing 10% LPDS for an additional 24 hours. Media was aspirated and cells were incubated with [^125^I]TYR^11^-Somatostatin 14 (Perkin Elmer) (0.3 nM in MEM +1% BSA for indicated times. Cells were washed with PBS, and counts per minute (cpm) were determined using a gamma counter (Perkin Elmer 2470 Wizard2 Automatic Gamma Counter).

### Radiolabeled Insulin Uptake Assay

Cells were plated in 96-well stripwell plates (1 × 10^4^ cells per well) in MEM with 10% FBS, 100 I.U./ml penicillin, 100 µg/ml streptomycin, 2mM L-glutamine, and 1X MEM non-essential amino acids. After 24 hours, media was removed and cells were incubated with MEM containing 10% LPDS for an additional 24 hours. Media was aspirated and cells were incubated with [^125^I] Insulin (0.8 ng/mL in MEM + 1% BSA) for 2, 5, or 10 minutes, which was followed by a PBS wash, and counts per minute (cpm) were determined using a gamma counter (Perkin Elmer 2470 Wizard2 Automatic Gamma Counter).

### DiI-LDL Uptake Assay

Cells were plated on 96-well plates (1 × 10^4^ cells per well) in MEM with 10% FBS, 100 I.U./ml penicillin, 100 µg/ml streptomycin, 2mM L-glutamine, and 1X MEM non-essential amino acids. After 24 hours, media was removed and then cells were incubated with MEM containing 10% LPDS for an additional 24 hours. Cells were washed 3 times in PBS then incubated with DiI-LDL (30 µg/mL) (L3482 Invitrogen) in MEM for 3 hours, followed by 3 washes with PBS. Images were acquired on a Carl Zeiss AxioObserver Z1 fluorescent wide field microscope.

### Filipin Cholesterol Detection

Cells were plated on 96-well plates (1 × 10^4^ cells per well) in MEM with 10% FBS, 100 I.U./ml penicillin, 100 µg/ml streptomycin, 2mM L-glutamine, and 1X MEM non-essential amino acids. After 24 hours, media was removed and then cells were washed 3 times in PBS and incubated with 50 µg/mL filipin (F4767 Sigma Aldrich, St. Louis, MO) in PBS for one hour in the dark at room temperature, followed by 3 washes with PBS. Images were acquired on a Carl Zeiss AxioObserver Z1 fluorescent wide field microscope. The signals were detected and quantified using Fiji, an image processing program based on ImageJ.

### Confocal Microscopy

Cells were plated in 8-well chamber slides (Lab-Tek cc2 plates, 154534PK Thermo Scientific) (5 × 10^4^ cells per well) in MEM with 10% FBS, 100 I.U./ml penicillin, 100 µg/ml streptomycin, 2mM L-glutamine, and 1X MEM non-essential amino acids. For the lipoprotein starved condition, media was removed after 24 hours then cells were incubated with MEM containing 10% LPDS for an additional 24 hours. For lipoprotein starved condition followed by LDL treatment, cells were then incubated with 50 µg/ml LDL in MEM for 3 hours. Cells were washed 3 times with PBS then fixed with 4% paraformaldehyde (Santa Cruz) for 10 minutes at room temperature, washed 3 times with PBS, then permeabilized with 0.1% Triton X-100 in PBS for 10 minutes at room temperature. Cells were blocked with 10% Goat Serum (50062Z Thermo Scientific) for one hour then incubated with goat anti-PGRMC1 (Abcam ab48012) 1:200 in PBST +1% BSA overnight, washed 3 times with PBST, then incubated with 1:250 donkey anti-goat Alexa488 secondary antibody (A-11055 Invitrogen) in PBST for 1 hour. Cells were then washed 3 times in PBST and blocked with 10% goat serum for an hour, then incubated with 1:50 mouse anti-LDLR (Novus NBP1-78159) in PBST +1% BSA overnight, washed 3 times with PBST, then incubated with 1:250 goat anti-mouse Cy3 secondary antibody (A10521 Invitrogen) in PBST for 1 hour. Cells were washed 3 times with PBST and blocked with 10% goat serum for an hour, then incubated with rabbit anti-TMEM97 primary antibody (Novus NBP1-30436) 1:200 in PBST +1% BSA overnight, washed 3 times with PBST, then incubated with goat anti-rabbit Alexa568 secondary antibody (A-11011 Invitrogen) 1:250 in PBST for an hour. Cells were washed 3 times in PBST, once in PBS, then mounted in Vectashield HardSet Antifade Mounting Medium with DAPI (H-1500 Vector Labs). Images were acquired at 40X magnification on a Leica STED 3X Super-resolution Confocal Microscope.

### Proximity Ligation Assay

For the Proximity Ligation Assay, the Duolink *In Situ* PLA Red kit was followed according to manufacturer instructions (Sigma DUO92105). Briefly, cells were plated in 8-well chamber slides (5 × 10^4^ cells per well) in MEM with 10% FBS, 100 I.U./ml penicillin, 100 µg/ml streptomycin, 2mM L-glutamine, and 1X MEM non-essential amino acids. For the lipoprotein starved condition, media was removed after 24 hours then cells were incubated with MEM containing 10% LPDS for an additional 24 hours. For lipoprotein starved condition followed by LDL treatment, cells were then incubated with 50 µg/ml LDL in MEM for 3 hours. Cells were washed 3 times with PBS then fixed with 4% paraformaldehyde (sc-281692 Santa Cruz) for 10 minutes at room temperature, washed 3 times with PBS, then permeabilized with 0.1% Triton X-100 in PBS for 10 minutes at room temperature. Cells were blocked for 1 hour at 37° with Duolink *In Situ* PLA Blocking Buffer (Sigma). Cells were then incubated overnight with the antibody pair PGRMC1 and TMEM97, or LDLR and TMEM97, or LDLR and PGRMC1 at the concentrations mentioned above. Cells were washed 3 times for 5 minutes with PLA Wash Buffer A (Sigma), followed by incubation with the two corresponding Duolink *In Situ* PLA Probes for 1 hour at 37°. This was followed by 3 washes with PLA Wash Buffer A, and incubation with 1X Ligase (Sigma) for 30 minutes at 37°. Cells were washed 3 times in PLA Wash Buffer A, then incubated for 100 minutes at 37° in Duolink Amplification buffer with 1X Polymerase. This was followed by 3 washes with 1X PLA Wash Buffer B, and 1 final wash with 0.01X PLA Wash Buffer B, mounting, and images were acquired at 40X magnification on a Leica STED 3X Super-resolution Confocal Microscope.

## Electronic supplementary material

Supplementary Figures and Captions
