# Supplementary material for: Sigma-2 Receptor/TMEM97 and PGRMC-1 Increase the Rate of Internalization of LDL by LDL Receptor through the Formation of a Ternary Complex
Source: Sci Rep. 2018 Nov 15;8:16845. doi: 10.1038/s41598-018-35430-3 (PMC6238005; doi:10.1038/s41598-018-35430-3)
Supplement: Supplementary file 1 — Supplementary Figures and Captions [file 41598_2018_35430_MOESM1_ESM.pdf]

Supplementary Information

Sigma-2 Receptor/TMEM97 and PGRMC-1 Increase the Rate of Internalization of LDL by LDL Receptor through the Formation of a Ternary Complex

Aladdin Riad, Chenbo Zeng, Chi-Chang Weng, Harrison Winters, Kuiying Xu, Mehran Makvandi, Tyler Metz, Sean Carlin, and Robert H. Mach

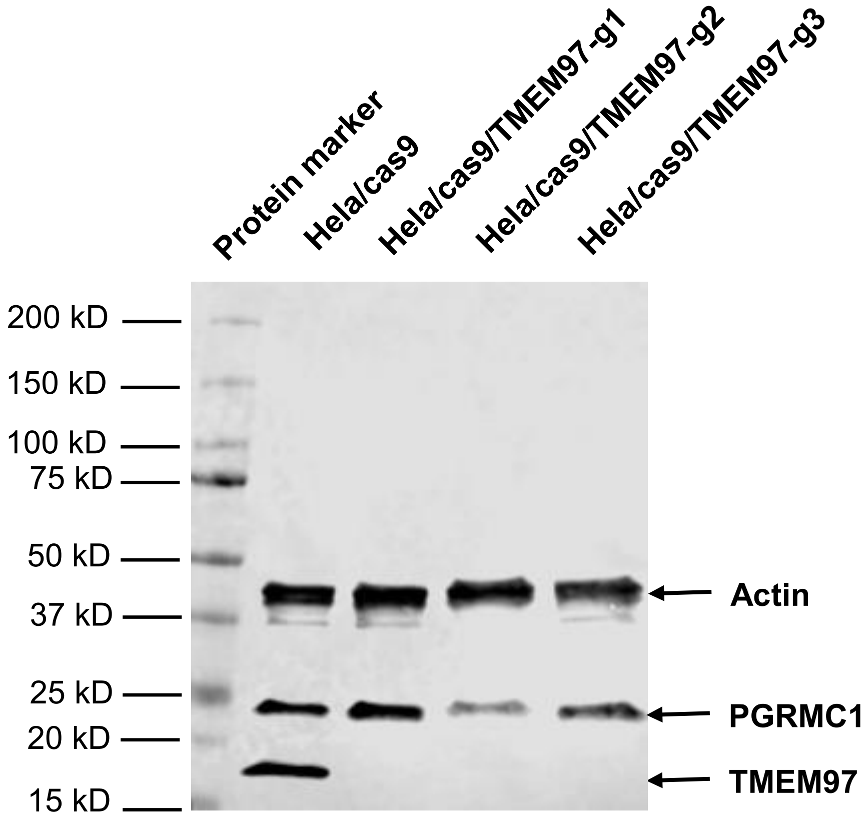

Supplementary Figure 1. Western Blots Analysis analysis indicated all sgRNA sequences ablated the presence of TMEM97 in HeLa cells.

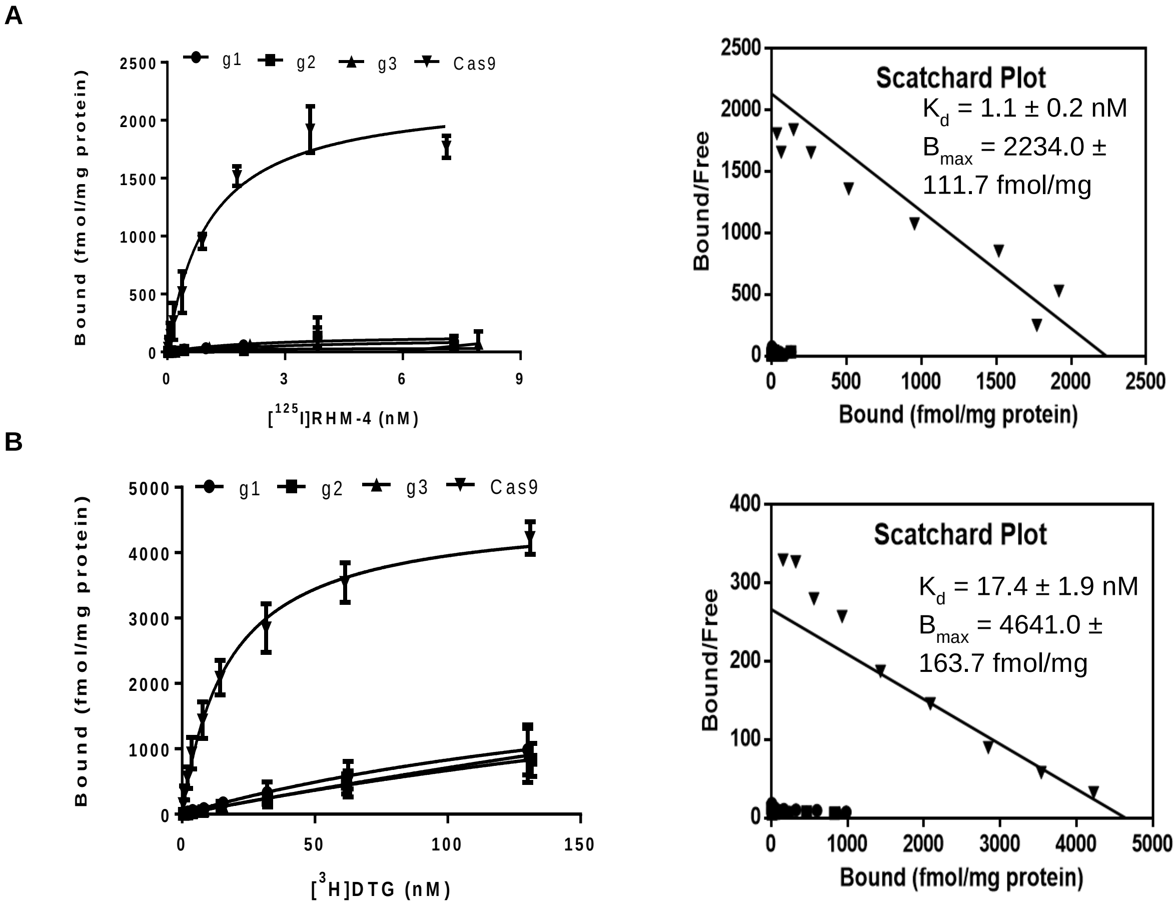

Supplementary Figure 2. Saturation binding curve and scatchard plots of (A) [^{125}]RHM-4 and (B) [^{3}H]DTG on three different clones of TMEM97 KO and control HeLa cell membranes. Experiments were conducted in duplicates, n=3.

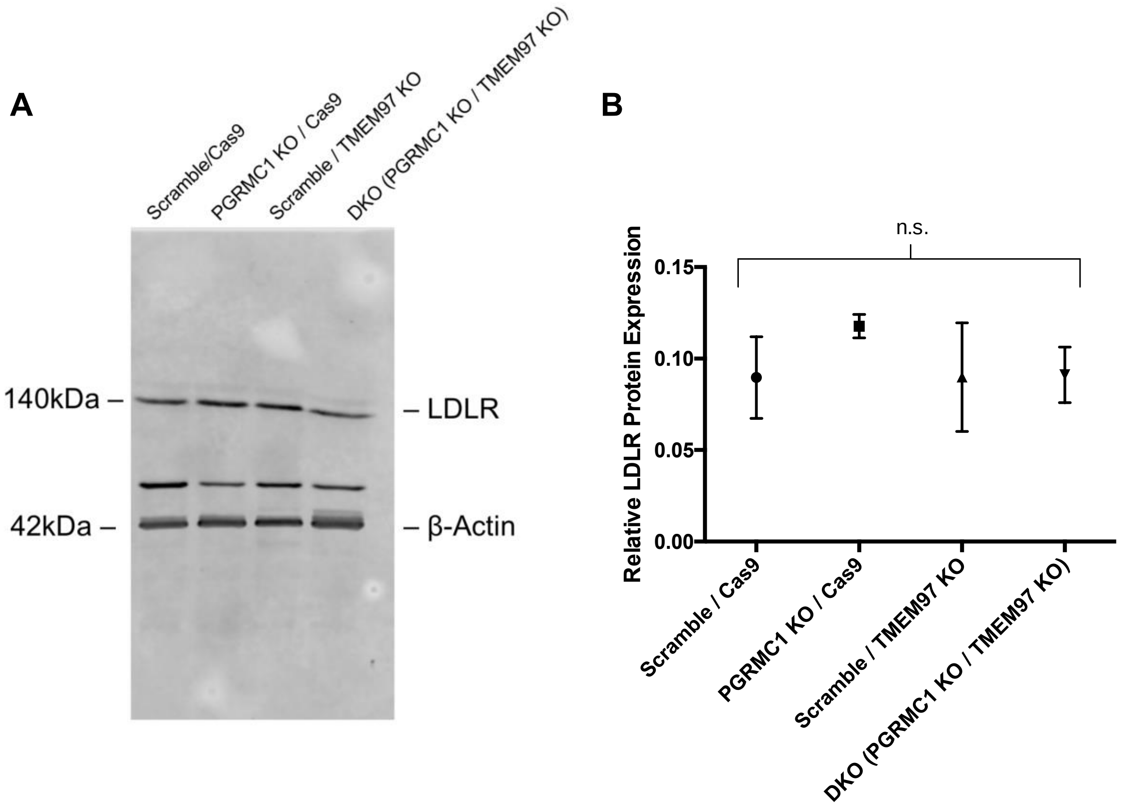

Supplementary Figure 3. Western Blots Analysis. Cells were sterol starved for 24 hours by incubation with lipoprotein depleted serum (LPDS) and 30µg/well of cell lysates were resolved on a 4-20% gradient gel. LDLR protein expression levels were measured and quantified as expression ratios relative to beta actin. There was significant difference in LDLR expression between the cell lines. Significance determined by a one-way Anova comparison between all cell lines, n.s. indicates not significant ($p>0.05$), n=3.

A

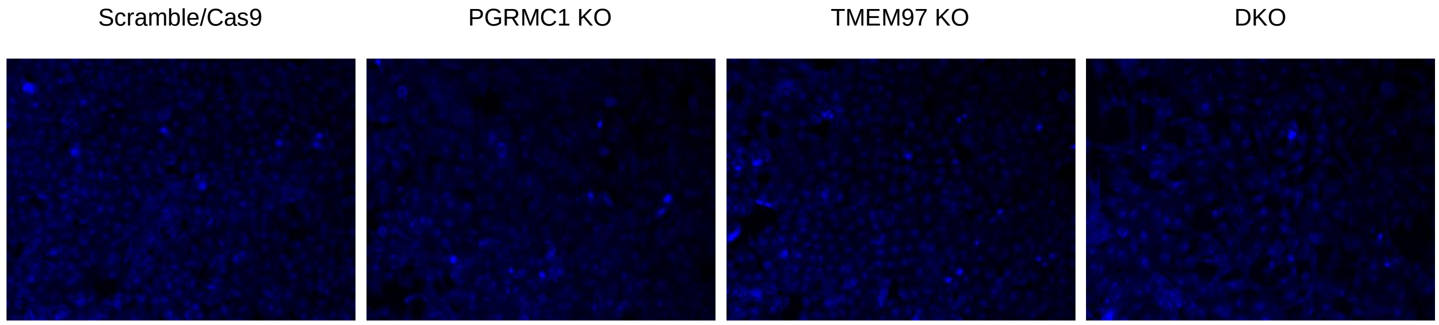

B

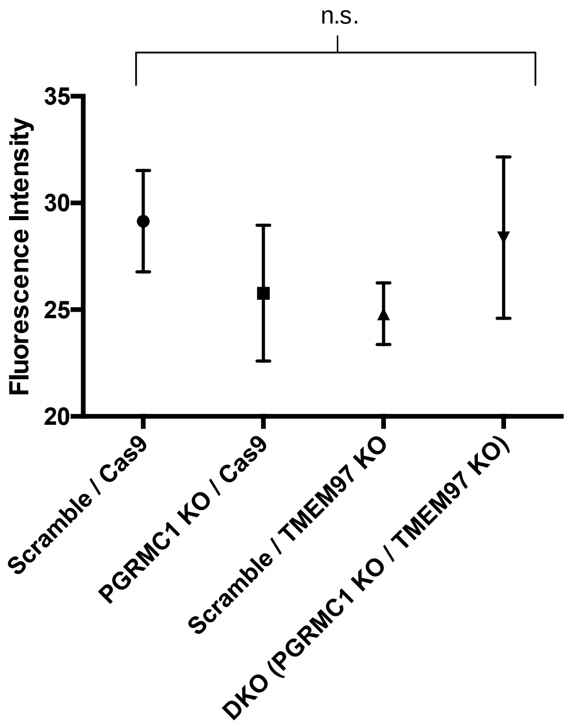

Supplementary Figure 4. Fluorescent signal of HeLa cell lines after Filipin staining for intracellular cholesterol. (A) Microscopy images and (B) quantified florescence intensity of staining. Significance determined by a one-way Anova comparison between all cell lines, n.s. indicates not significant (p>0.05), n=3.

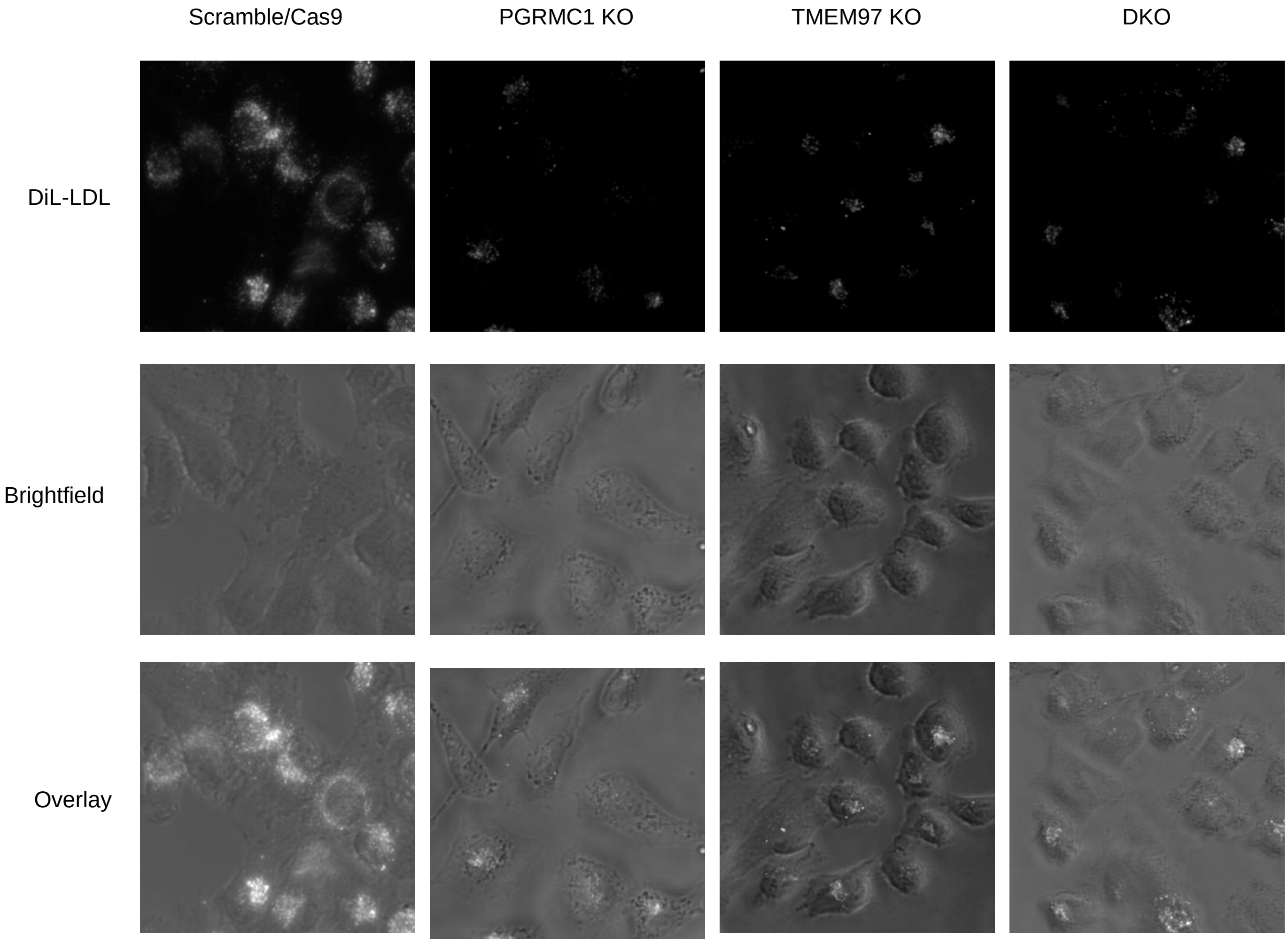

Supplementary Figure 5. Fluorescent signal, brightfield images of cells, and overlay of HeLa cell lines after 4 hour incubation with DiL-LDL.

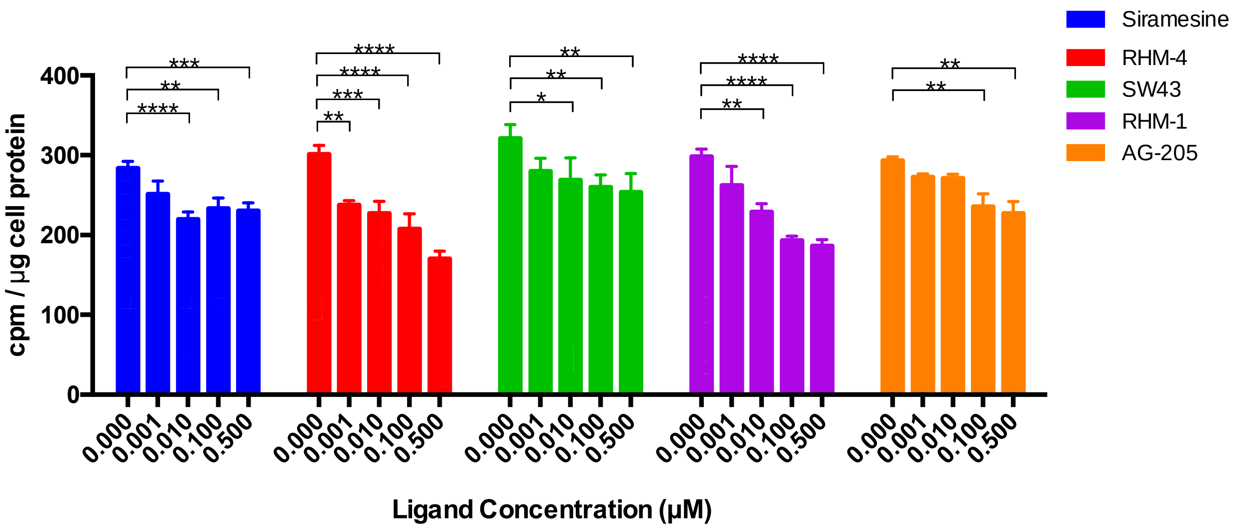

Supplementary Figure 6. The effect of various ligands on LDL uptake. Siramesine, RHM-4, SW43, RHM-1, and AG-205 were used at indicated concentrations. Significance determined by a 2-way Anova, * p<.05, ** p<.01, ***p<.001, ****p<.0001, n=3.

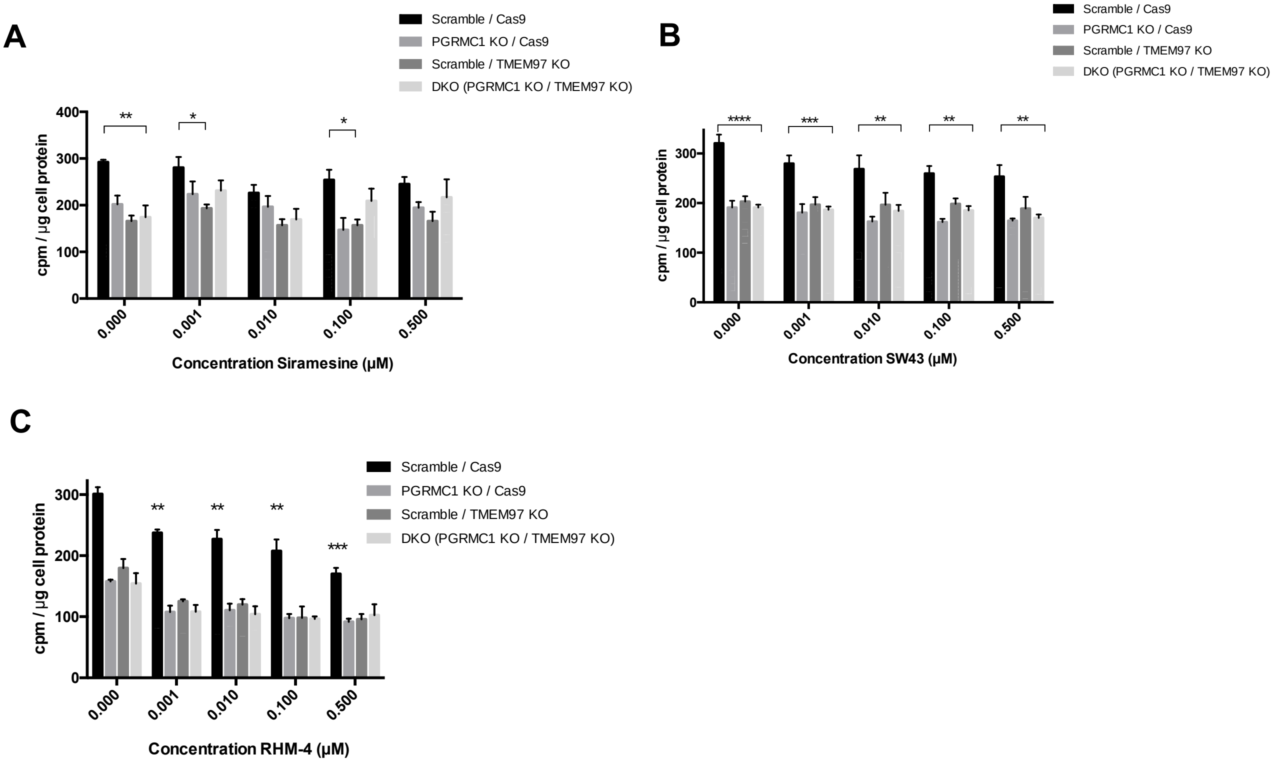

Supplementary Figure 7. The effect of various ligands on LDL uptake amongst all knockout cell lines. Siramesine, RHM-4, SW43 were used at indicated concentrations. Significance determined by a 2-way Anova, * p<.05, ** p<.01, ***p<.001, ****p<.0001, n=3.

A

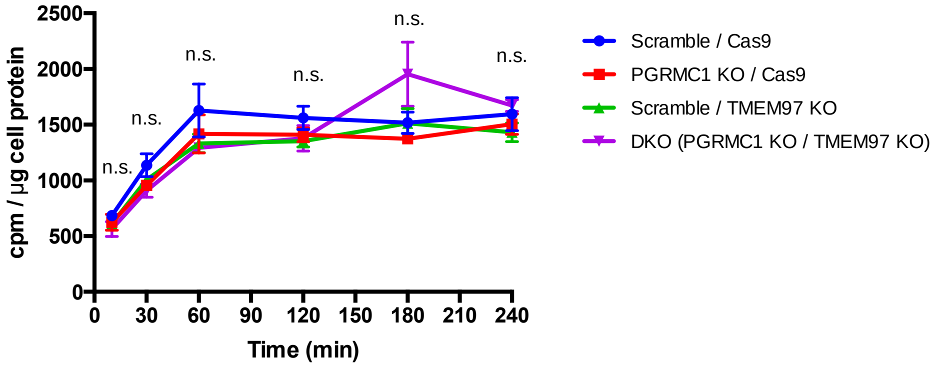

B

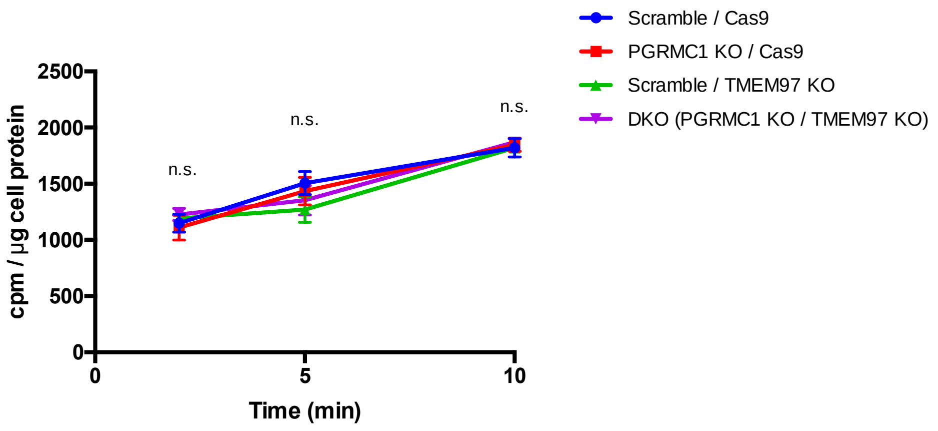

Supplementary Figure 8. Uptake over time of radiolabeled (A) [^{125}I]TYR^{11}-Somatostatin 14 (0.3nM) and (B) [^{125}I] Insulin (0.8ng/mL). Significance determined by a 2-way Anova comparison between cell lines, n.s. indicates not statistically significant ($p>0.05$), n=3.

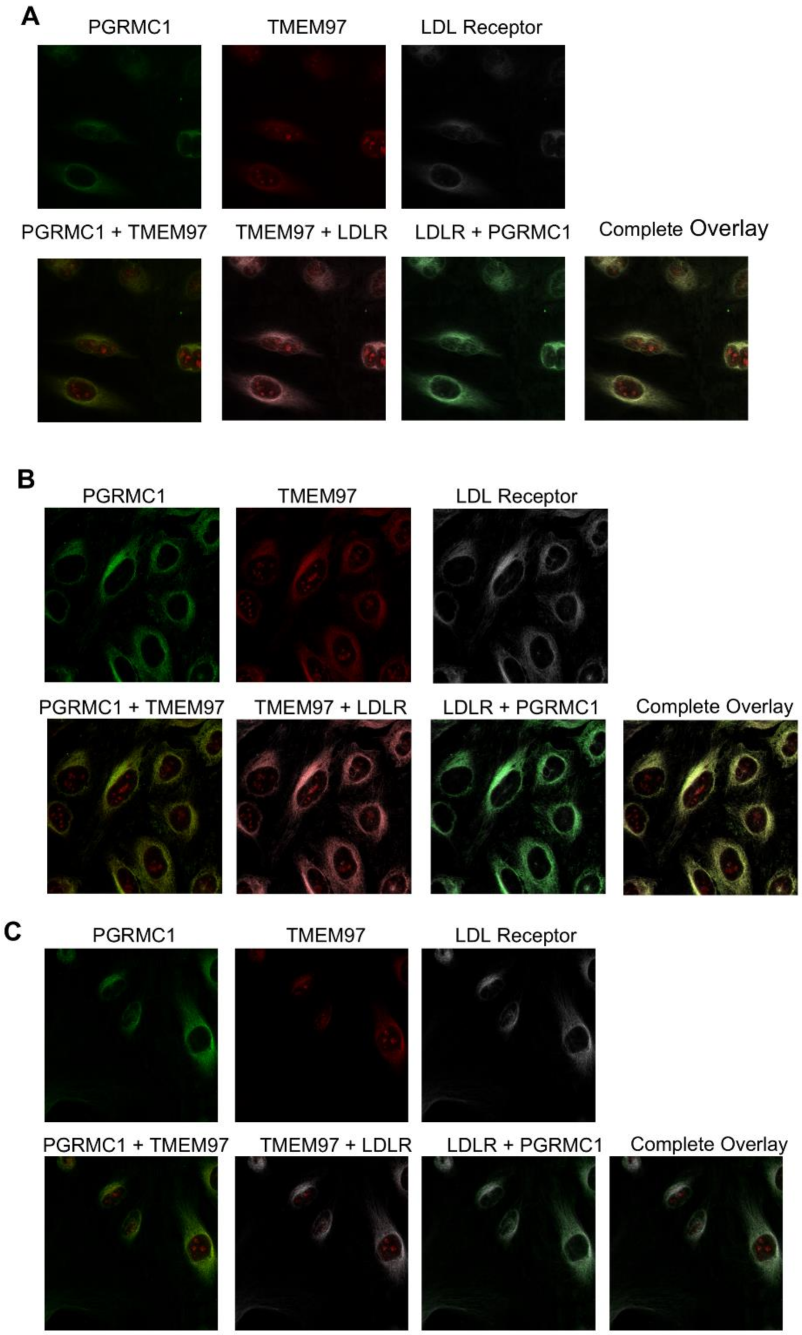

Supplementary Figure 9. Confocal microscopy on HeLa cell lines labeled with antibodies against LDLR, PGRMC1, and TMEM97. (A) Cells were plated and imaged under standard, non-lipoprotein depleted conditions. (B) Cells were plated and sterol starved by incubation with lipoprotein-depleted serum for 24 hours. (C) Cells were sterol starved for 24 hours, followed by treatment with 50 μg/mL LDL for 3 hours.

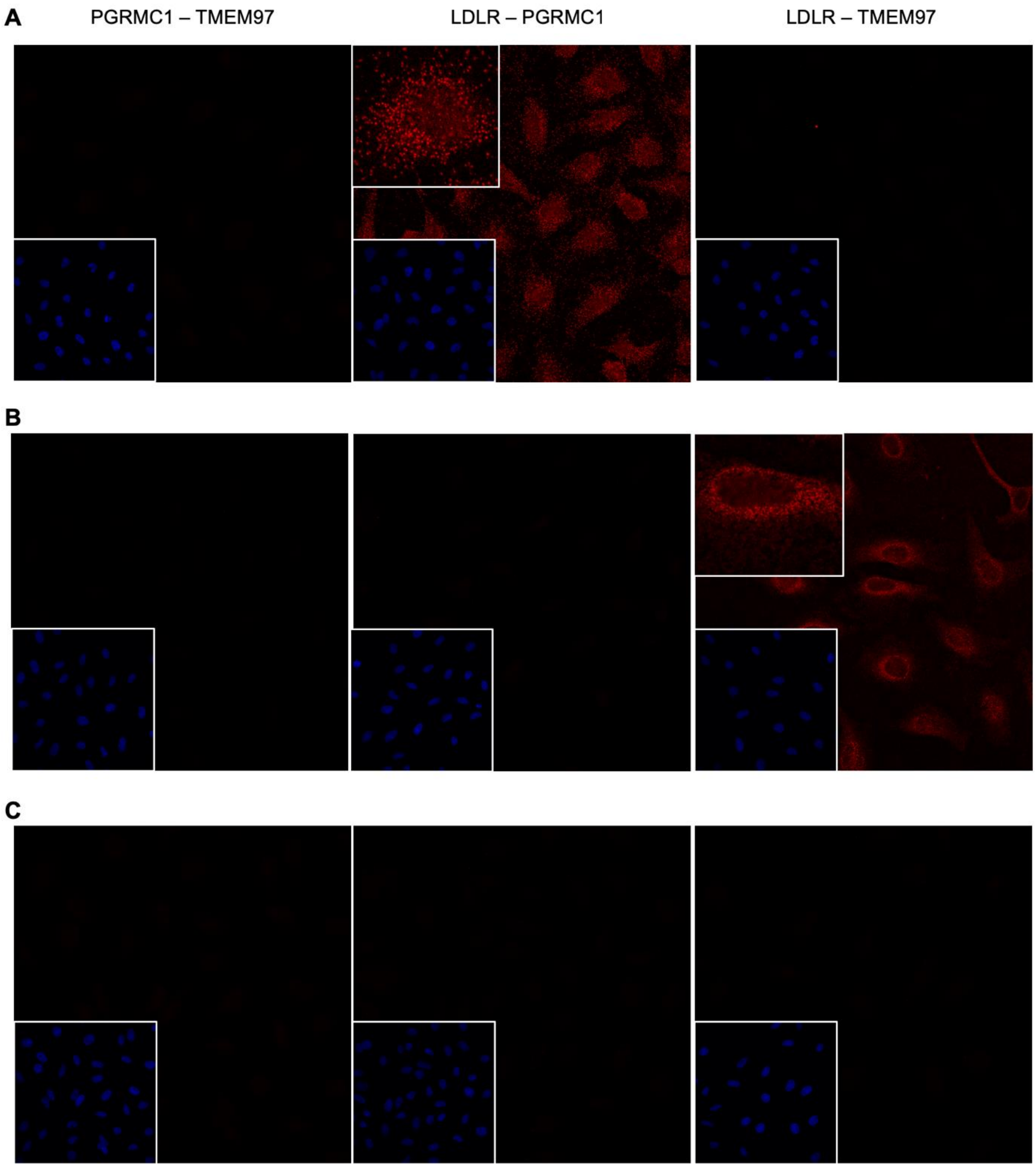

Supplementary Figure 10. Pairwise Proximity Ligation Assay on knockout cell lines. (A) Scramble / TMEM97 KO HeLa cells (B) PGRMC1 KO / Cas9 HeLa cells (C) Double knockout (TMEM97 KO / PGRMC1 KO). Inserts represent DAPI staining for the entire field (bottom), and where signal was detected a single cell was enlarged (bottom).
